# Divergent Genetic Pathways Underlying Convergent Parasitic Behaviours in Blowflies

**DOI:** 10.1111/mec.17785

**Published:** 2025-05-05

**Authors:** Gisele Antoniazzi Cardoso, Pedro Mariano‐Martins, Gustavo Amaral Faria, Inoka Karunaratne, Patricia Jacqueline Thyssen, Tatiana Teixeira Torres

**Affiliations:** ^1^ Department of Genetics and Evolutionary Biology, Institute of Biosciences University of São Paulo (USP) São Paulo São Paulo Brazil; ^2^ Laboratory of Integrative Entomology, Department of Animal Biology, Institute of Biology Universidade Estadual de Campinas (UNICAMP) Campinas São Paulo Brazil; ^3^ Department of Zoology, Faculty of Science University of Peradeniya Peradeniya Sri Lanka

**Keywords:** Calliphoridae, ectoparasitism, gene expression, larval behaviour, molecular evolution, oviposition

## Abstract

Blowfly (Diptera: Calliphoridae) exhibit diverse feeding strategies, with most species developing on decomposing organic matter. However, parasitism has evolved within the family, and some species convergently gained the ability to explore the tissues of living vertebrate hosts, which imposes critical veterinary, medical, and agricultural issues worldwide. It is yet unknown how this phenotype has evolved and whether it is determined by the same genetic architecture in different species or not. To address these questions, we evaluated key behavioural phenotypes in species with contrasting feeding habits, focusing on female oviposition preferences and larval survival on distinct diets, both critical aspects of their life cycles. These assays allowed us to propose hypotheses of how oviposition and larval behaviours contribute to feeding habits displayed in nature for parasitic and saprophagous species. Additionally, a transcriptome‐wide analysis revealed genes and functional pathways potentially linked to parasitic behaviour by comparing gene expression profiles and coding sequence evolution. In the genetic analysis, we identified genes with important functions related to the measured behaviours, revealing that distinct genes may underlie each independent case of parasitism evolution, suggesting a non‐parallel evolutionary pathway for this convergent trait.

## 1 Introduction

Species may exhibit similar traits as a result of shared ancestry, known as homologous traits (Hubbs 1944). However, evolution has also repeated itself throughout the history of life, leading to the independent evolution of similar traits. This phenomenon is known as convergent evolution, where traits are considered analogous (Hao et al. 2019; Hubbs 1944; Rosenblum et al. 2014). Analogy and convergence have been extensively studied at the organismal level (i.e., morphological and/or functional resemblance of structures present in different species). Classic examples include the independent evolution of wings in insects, birds, and bats, all of which lack common ancestry (Hubbs 1944). Convergent traits can also be observed at other levels of biological organisation, including complex behavioural and ecological patterns, such as eusociality in hymenopterans (Johnson et al. 2013).

Molecular data have also been widely used to study evolutionary convergence at the genetic level (Lamichhaney et al. 2019; Stern 2013). These studies often aim to understand whether the macroscopic phenotypic convergence observed in many species is underlain by a shared genetic pattern (see reviews by Hao et al. 2019; Rosenblum et al. 2014 and references therein). They also explore the distinction between parallel evolution, where the same genes and/or genetic pathways lead to similar traits, and non‐parallel evolution, where different genetic routes yield the convergent phenotype (Scotland 2011; Arendt and Reznick 2008).

The concepts of adaptation and constraint also play an important role in understanding evolutionary convergence. Losos (2011) explored how convergent traits can emerge due to similar selective pressures acting on unrelated species. However, he emphasised that phylogenetic constraints—the inherited characteristics of a lineage—may limit the evolutionary options available to a species, thereby shaping how and whether convergence occurs. This is important for investigations on the molecular basis of convergent evolution, as both selective pressures and genetic constraints may influence the paths available for species to evolve similar traits independently. This framework helps explain the broader patterns of convergence and divergence, as different species may be constrained by their evolutionary histories while adapting to similar ecological niches. Similarly, Elmer and Meyer (2011) discussed how convergent evolution can result from either the same or different genetic changes, depending on the ecological and genetic contexts. These considerations are essential for framing our research, which seeks to investigate whether convergent feeding behaviours, often associated with specific ecological pressures, share a common genetic basis or arise through distinct genetic pathways. By leveraging different approaches, we aim to uncover whether convergent feeding behaviours are driven by parallel genetic evolution (involving the same genes) or non‐parallel evolution (involving different genes) in a key family of flies.

The Calliphoridae (Diptera: Calyptratae), commonly known as blowflies, are a family within the Oestroidea clade (Kutty et al. 2019; Marinho et al. 2012; Rognes 1997; Yan et al. 2021) presenting a unique model to explore convergence at the intersection of behaviour and molecular evolution. Blowfly larvae require a protein‐rich food source for their development, typically feeding on animal tissues to meet nutritional requirements (Stevens 2003). Some species are obligate myiasis‐causing parasites, developing on living tissues of vertebrates, while others are saprophagous, feeding exclusively on decaying matter (Hall and Wall 1995; Stevens 2003; Thyssen et al. 2012). Facultative parasites are more versatile, as their larvae can feed on either living or dead tissues on a vertebrate host, or even on decaying corpses (Zumpt 1965; Hall and Wall 1995; Stevens 2003; Moretti and Thyssen 2006; Stevens and Wallman 2006; Thyssen et al. 2018). This classification, however, does not reflect the preferred substrate for larval feeding. Some species can be classified as facultative parasites even if they feed exclusively on dead tissues, provided they are physically on a host.

In addition to vertebrate parasites, some blowfly larvae have other trophic specialisations, including invertebrate parasitism and blood‐sucking behaviours (Stevens and Wallman 2006). In this study, however, we focus on species that cause myiasis in vertebrates. Specifically, species that feed exclusively on the tissues of a living vertebrate host, such as *Cochliomyia hominivorax* and *Chrysomya bezziana*, are major pests in the livestock industries of the New and Old World, respectively, causing significant economic losses (Hall and Wall 1995; Hall et al. 2009; Grisi et al. 2014; Scott et al. 2017). On the other hand, species that feed on decaying matter, such as *Cochliomyia macellaria* and *Chrysomya megacephala*, are commonly found in garbage dumps, carcasses, sewers, and exposed food in street markets. Occasionally, they can also be found feeding on necrotic tissues of living hosts (Hall and Wall 1995). Finally, some species are capable of feeding on both decaying and living host tissues, including *Chrysomya albiceps*, *Lucilia cuprina*, and *Lucilia eximia* (Muñoz‐Garcia et al. 2016; Bambaradeniya et al. 2023; dos Santos et al. 2024).

These diverse feeding habits of calliphorids do not strictly correlate with the evolutionary relationships within the family. For each obligatory parasite, there is at least one closely related saprophagous species (Stevens et al. 2006). It is believed that the calliphorid ancestor was saprophagous, with other feeding behaviours evolving later (Zumpt 1965). Under this hypothesis, parasitic myiasis must have evolved at least four times independently within the Calliphoridae phylogeny (Stevens 2003). This repeated evolution is not unexpected, as parasitism has independently arisen more than 223 times in animals, with 113 of these occurring between or within genera. The order Diptera alone accounts for at least 60 independent transitions from free‐living to a parasitic lifestyle (Weinstein and Kuris 2016).

Blowflies therefore hold substantial potential for the study of convergent phenotypes, particularly complex behaviours such as feeding habits. In this study, we aim to investigate the molecular basis of convergent feeding habits in blowflies by integrating behavioural assays and molecular analyses. To comprehensively understand the mechanisms underlying these transitions in Calliphoridae, two critical aspects involved in the evolution of larval feeding behaviour must be considered: the role of female oviposition site selection versus the role of larval ability to develop on specific substrates. Are females making active substrate choices that determine the feeding behaviour of their offspring, or are larvae limited by their ability to thrive on certain substrates? Moreover, we examine whether the convergence of parasitic feeding habits in *Co. hominivorax* and *Ch. bezziana* was driven by parallel genetic mechanisms—where the same genes and pathways are involved—or by non‐parallel mechanisms, where different genetic routes have led to similar ecological strategies. Given the important role of genes in shaping behavioural traits (Niepoth and Bendesky 2020), we hypothesise that blowflies with distinct feeding habits may exhibit genetic determinants associated with each lifestyle.

To address this, we employed transcriptome‐wide analyses to identify candidate genes and pathways involved in the evolution of parasitic feeding habits. The evolution of both coding and regulatory sequences has been linked to the evolution of phenotypic traits, including behaviours (Niepoth and Bendesky 2020), and traits exhibiting parallel or convergent evolution (Hao et al. 2019). By focusing on gene expression and sequence evolution, we aim to determine whether the convergence in feeding behaviour among blowflies has a shared genetic basis. The combination of molecular and behavioural data allows us to test whether the convergence in parasitic habits within blowflies has evolved by parallel genetic evolution or if it has arisen through distinct mechanisms.

## 2 Materials and Methods

### 2.1 Fly Collection and Rearing

We collected specimens throughout Brazil and established colonies for six calliphorid species: two species that feed exclusively on necrotic tissues (*Ch. megacephala*, and *Co. macellaria*), one obligate parasite (*Co. hominivorax*), and three facultative species that feed on both fresh and necrotic tissues (*Ch. albiceps*, *L. cuprina* and *L. eximia* ). Although *Co. macellaria*, *Ch. megacephala*, *Ch. albiceps*, *L. cuprina*, and *L. eximia* are all classified as facultative parasites due to their potential to infest vertebrate hosts, *Co. macellaria* and *Ch. megacephala* have not been observed feeding on healthy tissues. To reflect this distinction between the feeding preferences of larvae, we use the term “facultative” only for species capable of feeding on both dead and living tissues of vertebrates, while we refer to those species that do not invade healthy host tissues as “saprophagous”.

*Chrysomya albiceps*, *Ch. megacephala*, *Co. macellaria*, and *L. cuprina* were captured using an entomological net and/or a Van Sommeren‐Rydon trap with rotten bovine, chicken, or fish meat as bait. Larvae of *L. eximia* and *Co. hominivorax* were obtained from wounds of infested animals. Specimens of the obligate parasite *Ch. bezziana* were collected in Sri Lanka and reared until the adult stage, but no colony was established. The *Ch. bezziana* sample was collected under different conditions than the other species, which may have impacted our gene expression analyses. To mitigate potential biases, we included a *Ch. megacephala* sample from the same location, reared under similar conditions (Table S1).

Adult flies were kept in a climate chamber at 25°C (± 1°C) and 60% relative humidity (± 10%). Adults of all the species were fed a diet consisting of a mixture of sugar, whole milk powder, and brewer's yeast in equal proportions (1:1:1), along with *ad libitum* access to water. Facultative parasitic and saprophagous larvae were reared on decaying bovine meat at 25°C, while the obligate parasitic larvae were reared on fresh ground meat supplemented with water‐diluted blood (1:4) at 34°C. Upon reaching the prepupal stage, the larvae were allowed to pupate on sawdust and were kept at the same temperature as that used during rearing.

### 2.2 Larval Survival Assay

We assessed the survival of larvae on fresh and rotten bovine meat across six species (excluding *Ch. bezziana*). Larvae were reared exclusively on one of two substrates throughout the entire larval stage. One group was developed on rotten ground beef (decayed for 5 days at 25°C) at 25°C (± 1°C) and 40% relative humidity (± 10%). The second group was reared on fresh ground beef supplemented with 1% formalin and diluted bovine blood (1:2) at 37°C (± 1°C) and 80% humidity (± 10%). Bovine blood was obtained from a local abattoir and mixed with an anticoagulant (3.8% sodium citrate). The diluted blood is made up of 50% pure blood and 50% filtered water (Cunha et al. 2023). Larvae were allowed to pupate on sawdust, with pupae kept at the same temperature as the larval rearing environment until adult emergence. This approach differs from that described by Cunha et al. (2023), where pupae were maintained at 25°C in all cases.

For each species, 480 eggs were evenly distributed across three plastic vials (2.5 L) each containing 80 g of the respective substrate. Larval survival was determined by the number of emerged adults. To understand the specific behaviour of *Co. hominivorax*, larvae initiated their development in fresh diet+blood at 37°C, and then switched to rotten meat at 25°C after the second instar. The developmental rate was also measured by weighing *Co. hominivorax*’ larvae throughout their development until they reached the third stage. Every 12 h, 10 larvae were randomly sampled and weighed. For this species, the whole colony perished before we could perform the survival assay with the typical diet of the species (fresh ground beef supplemented with blood). Due to colony loss, we used survival data from Mastrangelo et al. (2014) which used a comparable diet of raw ground beef mixed with liquid diet containing spray‐dried red blood cells, spray‐dried egg, skimmed milk powder, and water.

### 2.3 Female Oviposition Site Preference Assay

To assess the preference for oviposition sites, we adapted the protocol from Spindola et al. (2016). In this assay, two substrates were offered for gravid females within a Y system (Figure S1): 1) a parasite‐preferred substrate consisting of fresh ground bovine meat supplemented with 1% formalin and diluted bovine blood (1:2), maintained at 32°C (± 2°C) using a heating pad—hereon referred to as fresh—and 2) a “saprophagous‐preferred” substrate, consisting of rotten bovine meat maintained at 25°C (the meat was previously decayed at room temperature for 48 h)–hereon referred to as rotten. Five females were released at the starting point and allowed to oviposit on either substrate for 3 h. Subsequently, the eggs oviposited on each substrate were counted. We conducted the experiments inside a chamber under the same conditions used for rearing adults (25°C and 60% humidity) and with a light on. The Y‐system was set up on shelves which were covered to avoid visual perception of any human by the flies.

For each replicate with eggs, we calculated a preference index (PI) using Equation (1) with slight modifications from Dworkin and Jones (2009), as described by Cunha et al. (2023). The PI for each species was the average of all replicates where oviposition occurred. PI values above 0.5 indicated a preference for parasite‐preferred substrate, whereas values below 0.5 indicated a preference for saprophagous‐preferred substrate. This assay was conducted in 20 independent trials per species, with the exception of *Co. hominivorax*, for which only eight trials were feasible. For the PI calculation, we considered only the trials in which eggs were laid as valid replicates.

(1) $$\mathrm{PI}=\frac{\#\text{eggs}\;\mathrm{on}\;\text{the fresh option}}{\#\text{eggs}\;\mathrm{on}\;\text{the fresh option}+\#\text{eggs}\;\mathrm{on}\;\text{the rotten option}}$$

### 2.4 RNA‐Seq

Total RNA was isolated from females and larvae from lab colonies with TRIzol Reagent (Invitrogen, Carlsbad, CA, USA) following the manufacturer's instructions. Following extraction, the samples were quantified with the Qubit 2.0 Fluorometer and Qubit RNA BR Assay Kit (Invitrogen, Carlsbad, CA, USA).

Each sample used for sequencing contained 1–2 μg of total RNA from two individuals (adult females or larvae). The RNA‐seq was outsourced to the sequencing facilities at Laboratório de Biotecnologia, ESALQ (University of São Paulo, Piracicaba, São Paulo, Brazil) and BGI Genomics sequencing center (Tai Po, Hong Kong) (Table S1). In the latter case, to prevent sample degradation during transportation, RNA was precipitated by the addition of absolute ethanol (2.5× sample volume) and 10% 3 M sodium acetate. The TruSeq mRNA protocol (Illumina, San Diego, USA) was used to prepare the libraries. They were sequenced using HiSeq 2500 equipment using the HiSeq SBS v4 kit (Illumina, San Diego, USA) with paired 100 bp or 150 bp reads.

### 2.5 Transcriptomes and Genomes

The quality of the reads was assessed using FastQC version 0.11.3 (Andrews 2010) and subsequently processed with Trimmomatic version 0.36 (Bolger et al. 2014) using the following parameters: LEADING:3 TRAILING:3 SLIDINGWINDOW:4:15 MINLEN:20. To optimise computational efficiency and the quality of assemblies, redundant sequences were collapsed.

We used the collapsed reads to assemble transcriptomes for five species lacking a reference genome (*Ch. albiceps*, *Ch. bezziana*, *Ch. megacephala*, *Co. macellaria*, and *L. eximia* ) with Trinity version 2.7 using default settings (Grabherr et al. 2011). The transcriptomes were assembled using a combination of the adult and larval samples. We also reassembled an additional transcriptome for *Oestrus ovis* (Diptera: Oestridae) to serve as an outgroup for ortholog prediction using reads from Cardoso et al. (2021). We removed gene isoforms using a Perl script provided by Trinity authors (get_longest_isoform_seq_per_trinity_gene.pl). We assessed the completeness of the transcriptomes using BUSCO version 3.0 (Simão et al. 2015) with the Diptera ortholog database.

We identified open reading frames in the transcripts using TransDecoder v5.5.0 (Haas et al. 2013). We also gathered publicly available genomic data for five Calliphoridae species ( *Calliphora grahami* , *Lucilia sericata*, *Chrysomya rufifacies Co. hominivorax*, and *L. cuprina*) and used the genome‐predicted coding sequences for downstream analyses (Table S2). As three independent assemblies are available for *Ch. rufifacies*, we concatenated the CDS from them and used cd‐hit‐est version 4.8.1 (Li and Godzik 2006) to remove redundant sequences.

### 2.6 Orthology Assessment

To identify shared orthologs among the species, we employed the pipeline developed by Yang and Smith (2014), which infers trees for each cluster of homologous sequences and removes potential paralogues. We adhered to the parameters recommended by the authors in nearly all steps. We customised the mafft_wrapper.py script to include the—adjustdirectionsaccurately parameter in the MAFFT v7.310 (Katoh and Standley 2013) command line, ensuring consistent orientation of all sequences within a cluster.

For paralog removal, we filtered the clusters using the Monophyletic Outgroups option. Each inferred tree was rooted using *O. ovis* as an outgroup, and the largest non‐duplicated monophyletic clade was retained. One‐to‐one ortholog trees were also kept. This process was applicable only to clusters containing four or more sequences; for those with fewer sequences, we manually filtered out duplicates. Subsequently, *O. ovis* sequences were excluded, and single‐sequence clusters were discarded. This resulted in 18,124 shared genes.

We annotated each cluster based on the *Co. hominivorax* sequence. We retrieved GO terms for this genome using eggNOG mapper v2.1.6 (Cantalapiedra et al. 2021). We also used eggNOG to annotate the transcripts in *Ch. bezianna*'s transcriptome with no homologue in *Co. hominivorax*.

### 2.7 Expression Analyses

We excluded orthologous sequences from species lacking RNA‐seq data (*Ca. grahami*, *Ch. rufifacies*, and *L. sericata* ), retaining clusters with at least two remaining sequences (*n* = 13,370). We performed multiple‐sequence alignments with MAFFT v7.310 (Katoh and Standley 2013) and then removed gaps in the beginning and end of each alignment. We selected all clusters whose aligned sequences were at least 300 bp long and, within each selected cluster, removed all gaps, and individually excluded unaligned sequences less than 300 bp.

Afterwards, we realigned the remaining sequences within each cluster and performed a second step of gap removal. We used this final set of orthologues for gene expression quantification. RNA‐seq read counts (for both larvae or female data) were generated using Salmon v1.5.2 (Srivastava et al. 2020).

We identified differentially expressed genes (DEGs) using DESeq2 (Love et al. 2014), by comparing each obligate parasite to a congeneric non‐parasitic counterpart (i.e., *Co. hominivorax* vs. *Co. macellaria*, and *Ch. bezziana* vs. *Ch. megacephala*). We intersected the results to identify genes with convergent variations in gene expression. The motivation behind these comparisons stems from the hypothesis that if gene expression changes between parasitic and non‐parasitic species are similar in both *Cochliomyia* and *Chrysomya* genera, we could identify parallel gene expression shifts that may underlie the evolution of parasitism. Broader analyses of gene expression differences including the remaining species were not conducted to avoid complications arising from phylogenetic dependency.

We identified enriched GO terms in the overlapping sets using *Co. hominivorax* genome as the background. Enriched terms were also assessed in the complete results from each pairwise comparison, using either the *Co. hominivorax* genome or the *Ch. bezziana* transcriptome as backgrounds, overlapping the enriched terms afterwards. In all cases, we used GOATOOLS (Klopfenstein et al. 2018) and corrected *p*‐values using the false discovery rate (FDR) with a threshold of 0.05.

### 2.8 Correlation of Expression and PI

We converted female TPM (transcripts per million) expression values into TPM10K ($\mathrm{TPM}10K=\frac{\mathrm{TPM}\times N}{10^{4}}$, in which *N* represents the reference size for the species—in our case, *N* is the number of orthogroups that included said species—*sensu* Munro et al. 2022) to facilitate the comparison across libraries from different species. We selected genes expressed in all species (*n* = 4832) and calculated the median value for each gene in each species. We correlated these values to logit transformed PI values ($\ln\frac{\mathrm{IP}+1}{2-\mathrm{IP}}$).

Since no PI measurement was available for *Ch. bezziana* and considering the absence of evidence of this species in a non‐parasitic context, we reasonably assumed its PI to be equal to that of *Co. hominivorax* due to their similar ecological habits.

We tested the correlations using a phylogenetic‐informed approach with the *caper::pgls* function (Orme 2013) and the tree from Marinho et al. (2012). We considered all models with *p*‐value < 0.05 after FDR correction.

### 2.9 Evolution of Coding Sequences

We selected orthologous clusters with five or more sequences containing both, *Ch. bezziana* and *Co. hominivorax* (*n* = 6038) for further analysis, due to their parasitic habit. We performed codon‐wise alignments in each of them using MAFFT v7.310 (Katoh and Standley 2013), implemented in TranslatorX (Abascal et al. 2010). We edited the TranslatorX script to add the—adjustdirectionsaccurately option for MAFFT.

We conducted molecular evolution tests using CodeML (Yang 2007), implemented in the ETE Toolkit v3.1.2 (Huerta‐Cepas et al. 2016), employing the tree topology from Marinho et al. (2012). We ran branch (b_free) and branch‐site (bsA) models, along with their respective null models (b_neut and M0; and bsA1 and M1) for each cluster, with either *Co. hominivorax* or *Ch. bezziana* designated as foreground. We selected the best fit models using LRTs (likelihood ratio tests), and *p*‐values were corrected for multiple testing using FDR with a threshold of 0.05.

We considered the genes (and processes) with shifts in selection regime in both parasitic species as candidates for involvement in the convergent evolution of obligate parasitic behaviour. Following the approach used for the DEG sets, we adopted two strategies. First, genes with significant results identified in each species were overlapped, and the resultant set was used for GO enrichment analysis using GOATOOLS (Klopfenstein et al. 2018), with *Co. hominivorax*'s genome as the background. Second, we searched for enriched terms among the significant results of *Co. hominivorax* and *Ch. bezziana* separately and then overlapped the enriched terms.

## 3 Results

### 3.1 Larval Survival

**FIGURE 1:**
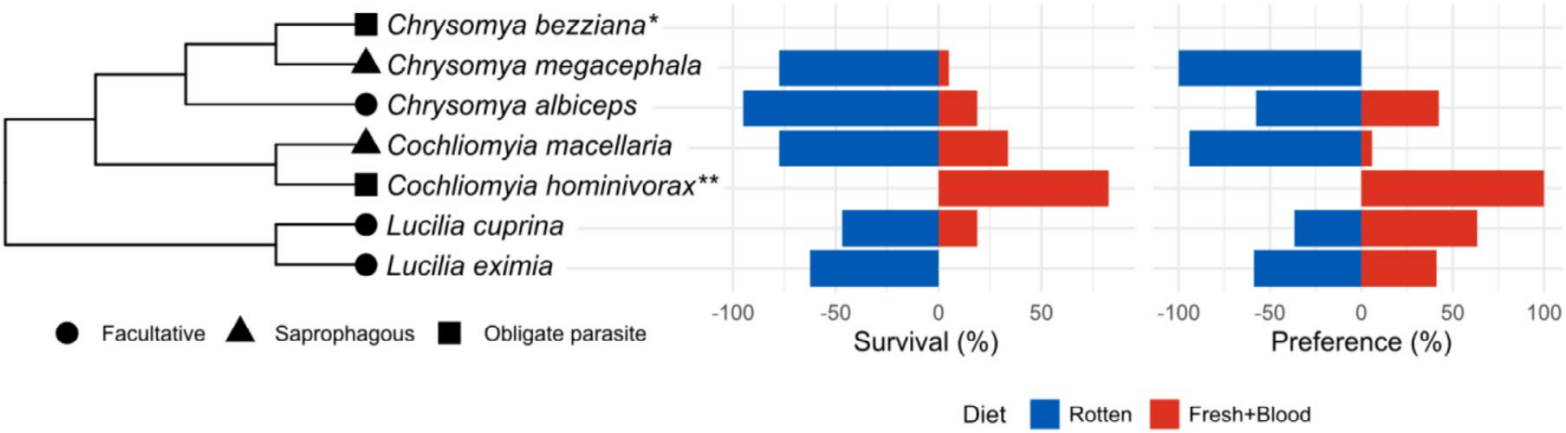
Comparative larval survival and female oviposition preference of blowfly species. The tree depicts the relationships among the seven blowfly species, with each species labelled by a symbol representing its ecological habit: Squares for obligate parasites, triangles for saprophagous species, and circles for facultative parasites. Adjacent to the tree, histograms illustrate the larval survival (left) and oviposition preference (right) of each species on two diets: Fresh+Blood at 37°C (red) and Rotten at 25°C (blue). Preferences and survival rates are shown as percentages, with positive values indicating Fresh+Blood at 37°C and negative values representing Rotten diet at 25°C outcomes. The obligate parasitic species (*Co. hominivorax*) fails to develop on the Rotten diet, while facultative and saprophagous species survive on both substrates. Female oviposition preferences further differentiate facultative from saprophagous species, with facultative species ovipositing frequently on both diets. * Assays with *Ch. bezziana* were not performed. ** *Co. hominivorax* data comes from Mastrangelo et al. (2014).

We first recorded larval survival rates of six species on different diets (fresh at 37°C and rotten at 25°C meat), observing a stark contrast between the obligate parasitic *Co. hominivorax* and the other species. While *Co. hominivorax* larvae exhibited almost no survival on the rotten 25°C diet—failing to progress beyond the first instar—all other species thrived on this diet. Survival rates on the rotten 25°C diet were high across facultative and saprophagous species, with at least 78% of eggs developing into adult flies (Table 1, Figure 1). In contrast, survival on the fresh 37°C diet was lower, ranging from 0% to 34%, with *Lucilia eximia* failing to reach the pupal stage. Adult flies of the other facultative and saprophagous species emerged on both fresh at 37°C and rotten at 25°C diets.

**TABLE 1:** Values of larvae survival and female preference index for each species.

Species	Larvae survival (%)		Female preference index (PI)		
	Fresh (37°C)	Rotten (25°C)	PI mean	PI standard deviation	PI logit transformation
*Ch. albiceps*	19.17	95	0.424	0.475	−0.1020835
*Ch. megacephala*	5.42	82.92	0.000	0.000	−0.6931472
*Co. hominivorax*	92.7^a^	0	1.000	0.000	0.6931472
*Co. macellaria*	33.75	77.5	0.059	0.243	−0.6061358
*L. cuprina*	0	96.25	0.634	0.443	0.1785892
*L. eximia*	0	78.33	0.412	0.497	−0.1176817

^a^ Data from Mastrangelo et al. (2014).

Most *Co. hominivorax* eggs did not hatch on the rotten diet (Figure S2). The few surviving larvae (approximately 10) perished within the first 12 h, failing to reach the third instar. Given *Co. hominivorax*'s apparent inability to develop on the decaying diet, we conducted a switch assay: eggs hatched on the fresh diet at 37°C, and larvae consistently gained weight until 36 h post‐hatching, when they were switched to the rotten diet at 25°C. Twelve hours after the switch, larval weight dropped from 0.004 g to 0.003 g (Figure S2, Table S3). Some larvae migrated above the diet and onto the container, contrasting with their typical behaviour of burrowing into the substrate (Figure S2). All larvae died 60 h after the switch. The dead larvae exhibited no traces of food in their gut, indicating that they had stopped feeding (Figure S2). These observations confirm the strict dietary requirement of *Co. hominivorax* for fresh tissue at high temperatures, as all larvae died without reaching the third instar.

### 3.2 Oviposition Site Preference

Subsequently, we examined female oviposition preferences to further understand the different habits observed in nature. A high rate of replicates exhibited no oviposition activity. For instance, females of *Ch. megacephala*, *L. eximia* , and *Co. hominivorax* laid no eggs in twelve (60%), eleven (55%), and four (50%) replicates, respectively (Table S4). The saprophagous species *Co. macellaria* and *Ch. megacephala* exhibited a marked preference for rotten meat at 25°C, with average PI values of 0.059 and 0, respectively (Table 1). In contrast, as anticipated, the obligate parasite *Co. hominivorax* demonstrated a strong preference for the fresh diet at 37°C, with a PI of 1, indicating exclusive oviposition on this substrate.

The results for facultative parasites were remarkably consistent. Females laid eggs on both diets, occasionally within the same replicate. *Lucilia cuprina* had a mean PI value of 0.634, showing a slight preference for the fresh diet at 37°C. Conversely, *Ch. albiceps* and *L. eximia* had a mean PI value of 0.424 and 0.412, respectively, with females showing a slight preference for the rotten diet (Table 1).

### 3.3 Expression Analyses

RNA‐seq data were generated for five blowfly species, with read lengths of 100 or 150 bp across multiple replicates and locations. The total number of raw reads per sample ranged from 14.7 million to 52.3 million, with the highest sequencing depth observed in *Co. hominivorax* larvae (52.3 million reads) (Table S1). The raw reads were assembled, resulting in varying numbers of transcripts across species, ranging from 62,922 to 284,557 (Table S5). We evaluated the completeness and quality of these transcriptomes using BUSCO. Among all transcripts, *Ch. albiceps* and *L. eximia* had the highest percentages of complete BUSCOs (94.28% and 94.53%, respectively), while *Ch. bezziana* had the lowest (87.92%). *Cochliomyia macellaria* , *L. eximia* , and *Ch. megacephala* showed a very high duplication rate (76.46%, 66.02%, and 61.74%, respectively) in the full transcript set, which dropped significantly to 23.01%, 0.29%, and 3.07% when only the longest isoform was analysed (Table S5). We used this latter dataset for the subsequent analyses.

**FIGURE 2:**
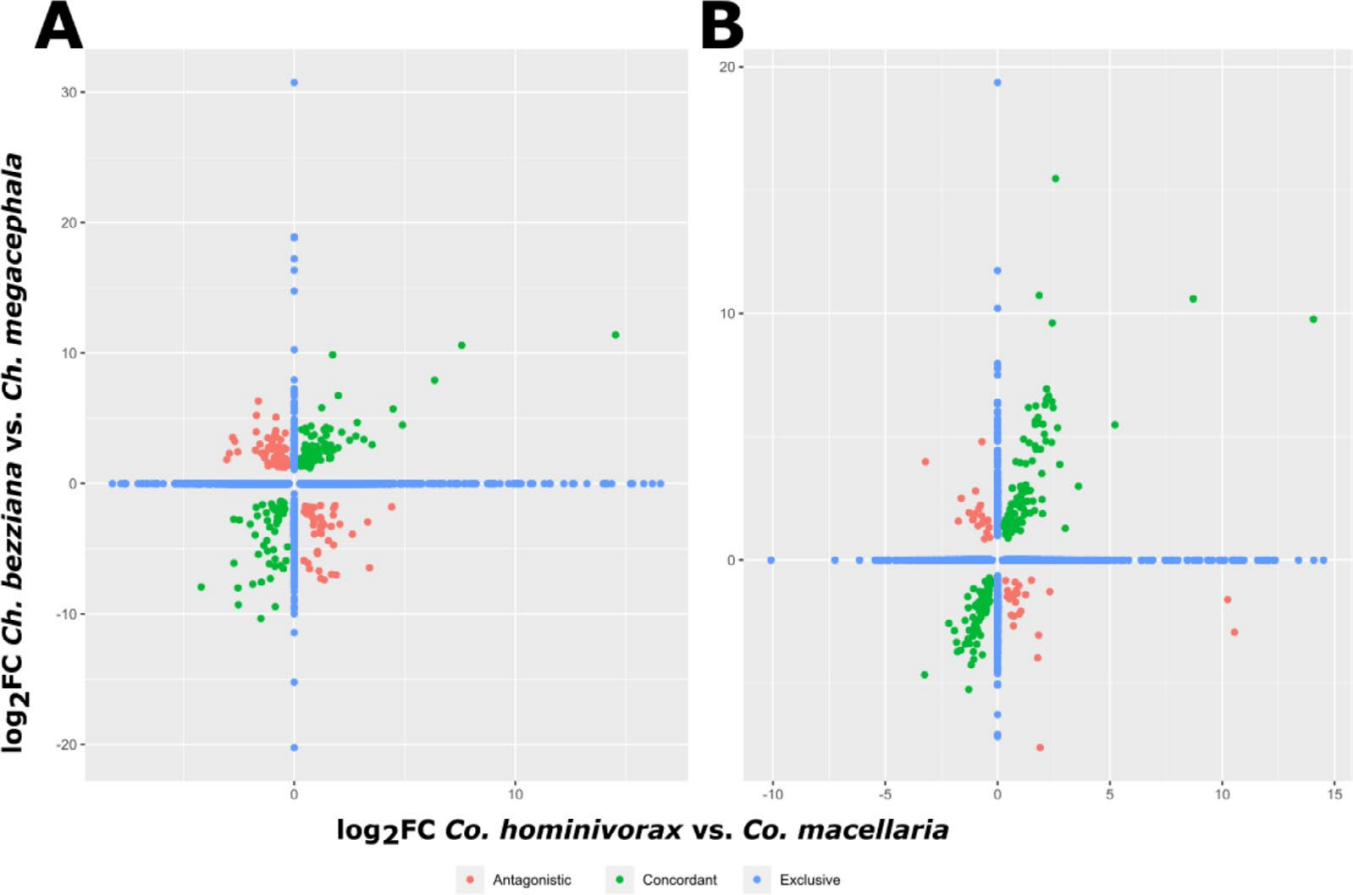
Differentially expressed genes in the comparisons between congeneric species with different feeding preferences. The expression fold change of genes is shown for *Co. hominivorax* vs. *Co. macellaria* and *Ch. bezziana* vs. *Ch. megacephala* in larvae (A) and female (B) RNA‐seq datasets. Positive values indicate higher expression in the obligate parasites (*Co. hominivorax* or *Ch. bezziana*), while negative values indicate lower expression. Genes are categorised as: (1) **antagonistic genes**, which are differentially expressed in both comparisons but regulated in opposite directions in each obligate parasite; (2) **concordant genes**, which are differentially expressed and regulated in the same direction in both comparisons; and (3) **exclusive genes**, which are differentially expressed in only one of the comparisons (log_2_FC = 0 in the comparison between species of the other genus).

In line with the behavioural observations, we analysed differential expression across species to explore whether parasitic and non‐parasitic species exhibit distinct expression patterns across the larval and adult stages that would correlate with their preferred diet. Out of the 18,124 orthogroups, we identified 2963 differentially expressed genes (DEGs) in the comparison between larvae of congeneric *Cochliomyia* species and 2027 between females (Table S6). For *Chrysomya*, we found 594 DEGs comparing the larvae of the two different species and 558 comparing the females (Table S7). Intersecting the results from both comparisons revealed 246 and 214 common DEGs in larvae and females of different genera, respectively (Tables S8 and S9). Among these shared DEGs, a subset exhibited consistent expression patterns between the obligate parasitic species in both genera—up‐ or down‐regulated in both obligate parasites (hereon termed concordant genes). Meanwhile, other genes showed opposing expression directions in obligate parasitic species across genera, suggesting opposing regulation (hereon termed antagonistic genes) (Figure 2; Table 2).

**TABLE 2:** Number of overlapping DEGs. The number of over‐ or underexpressed genes is relative to the obligate parasitic species. Concordant genes are those that are either over‐ or underexpressed in both parasitic species compared to their respective congeneric species. Antagonistic genes are overexpressed in one parasitic species but underexpressed in the other.

Comparison	Intersection (total)	Concordant		Antagonistic
		Overexpressed	Underexpressed	
Larvae	246	78	55	113
Females	214	97	71	46

To assess whether gene expression patterns in *Co. hominivorax* and *Ch. bezziana* exhibit more convergence than expected by chance, we compared the observed number of differentially expressed genes (DEGs) shared between both species to an expectation under an independence model. If gene expression evolved independently in each lineage, the probability of a gene being differentially expressed in both comparisons (*Co. hominivorax* vs. *Co. macellaria* and *Ch. bezziana* vs. *Ch. megacephala*) should be the product of the independent probabilities of being a DEG in each comparison.

Our results indicate a significantly higher number of genes exhibiting concordant expression patterns (upregulated or downregulated in both obligate parasites) than expected under this model (Table S10). In females, the expected number of concordantly upregulated genes was 28, yet 97 were observed (*χ* ^2^ = 170.17; *p* < 0.0001), and for concordantly downregulated genes, 24 were expected while 71 were observed (*χ* ^2^ = 91.97; *p* < 0.0001). For the larvae, we had significantly more concordantly upregulated genes (*χ* ^2^ = 41.85; *p* < 0.0001) but not downregulated genes (*χ* ^2^ = 2.6; *p* = 0.10).

Conversely, we did not observe a significant deviation from the expectation in the number of antagonistically expressed genes (upregulated in one obligate parasite and downregulated in the other) in females (*χ* ^2^ = 0.86; *p* = 0.35). However, larvae showed a moderate excess of antagonistically expressed genes (*χ* ^2^ = 11.57; *p* < 0.001), suggesting some degree of divergent regulation between *Co. hominivorax* and *Ch. bezziana*.

While these results support a degree of convergence in gene expression among obligate parasites, it is important to consider potential confounding factors. One key factor is covariation in gene expression due to shared regulatory mechanisms, which could inflate the observed number of concordantly differentially expressed genes. If certain functional groups of genes tend to be co‐expressed across species due to common regulatory constraints, this could lead to an excess of concordant DEGs even in the absence of true convergent evolution. Functional constraints may also contribute to the observed patterns, as genes involved in similar physiological and metabolic adaptations could be co‐regulated across species, regardless of independent evolutionary origins.

The limited number of concordant genes in the intersections precluded GO enrichment analysis. Alternatively, we identified enriched terms for the up‐ and down‐regulated genes separately in each congeneric comparison and then overlapped the results. This approach allowed us to identify 203 commonly enriched GO terms among up‐regulated genes and 246 among down‐regulated genes in parasitic larvae. These terms were associated with immune response, signalling, response to stress, response to external stimuli and, predominantly, development; either among genes that were up‐ or down‐regulated in the parasitic species. Additionally, terms associated with the regulation of transcription and translation were enriched in the up‐regulated overlapping gene set (Table S11). In females, most terms (394 among up‐regulated genes, and 104 among down‐regulated genes) were associated with the control of gene expression and sexual reproduction (Table S12).

We also performed a correlation analysis to explore the relationship between gene expression and preference index (PI), which reflects female preference towards fresh meat at high temperatures and mirrors the trophic specialisations and classifications of the species studied here. We identified 12 genes whose expression was significantly correlated with species' PIs, showing high *R* ^2^ values (> 0.95) and predominantly negative slopes, indicating lower expression in species with higher PI (Table 3; Figure S3). Among these, 10 genes were differentially expressed between *Co. hominivorax* and *Co. macellaria* and one between *Ch. bezziana* and *Ch. megacephala* females in the previous analysis.

**TABLE 3:** Genes with significant correlation between gene expression and the preference index as calculated in the adult female oviposition choice assay.

Ortholog in *Co. hominivorax*	Annotation	Adjusted *R* ^2^	Intercept	Slope
g4938.t1	CG4078	0.989081	41.01467	−32.9911
g20489.t1	Dystroglycan	0.98908	54.83671	12.40605
g16647.t1	meso18E	0.983573	25.37245	−11.1114
g20205.t1	prominin‐like	0.971956	12.16261	−5.94266
g17844.t1	Der‐2	0.969813	165.9868	−157.699
g17763.t1	Cpr51A	0.953368	42.04325	−9.07673
g8191.t1	CG6745	0.963109	75.65901	−73.7224
g6972.t1	Zizimin‐related	0.956315	20.98001	−13.7692
g520.t1	Pak3	0.953404	14.89588	5.322416
g3973.t1	CG6428	0.960846	17.27601	9.950748
g13439.t1	CG1317	0.956015	7.51365	9.104803
g562.t2	uninflatable	0.952407	71.15928	−45.8968

### 3.4 Evolution of Coding Sequences

**FIGURE 3:**
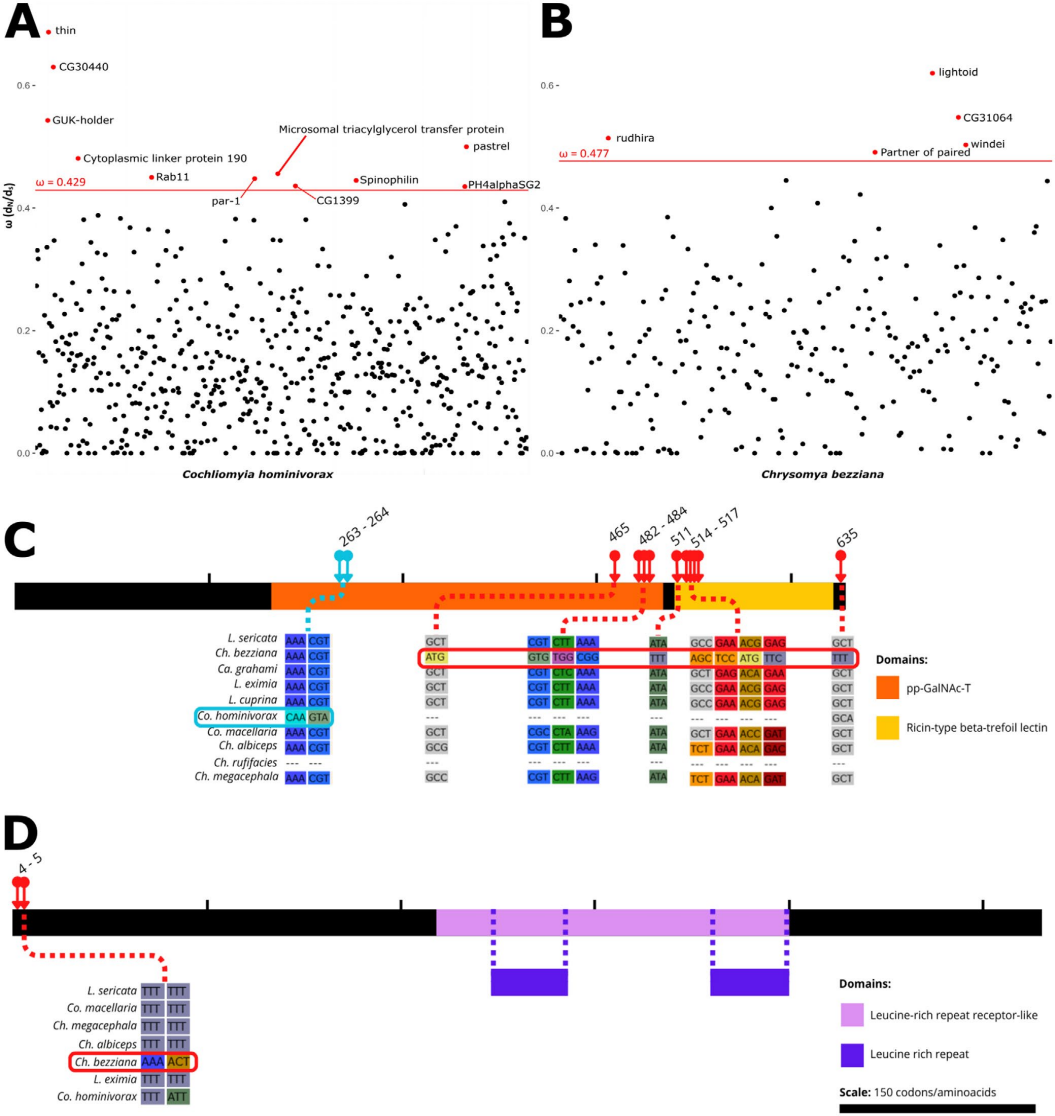
Coding sequence evolution. (A, B) Distribution of ω values for genes for which *b_free* as the best fit in branch tests with *Co. hominivorax* (A) or *Ch. bezziana* (B) as foreground species. Each dot represents a gene; red dots represent genes with ω values exceeding the outlier threshold (indicated by the red line). (C, D) Two examples of branch‐site results: Pgant5 (C) and Tollo (D). Both are depictions of the proteins encoded by these genes. Black stripes represent the proteins, whilst coloured sections indicate functional domains identified with *NCBI Conserved Domains* tool. Light‐blue arrows indicate sites under positive selection on the *Co. hominivorax* branch, and red arrows mark those on the *Ch. bezziana* branch. For each of these sites, codon alignments across species are shown, with the foreground species' sequence highlighted with a rectangle (light‐blue—*Co. hominivorax*—and red—*Ch. bezziana*).

To identify genes potentially involved in the evolution of parasitism, we assessed changes in selective pressures across branches, focusing on *Co. hominivorax* and *Ch. bezziana*. Our aim was to uncover genes under differential selection regimes in both species and identify shared or species‐specific adaptations associated with their obligate parasitic lifestyles. In the branch tests, we found 617 genes for which the *b_free* model was the best fit with *Co. hominivorax* as the foreground species (Table S13) and 266 with *Ch. bezziana* (Table S14). In *Co. hominivorax*, 489 genes were evolving faster, and 128 were evolving slower than the background. For *Ch. bezziana*, 218 genes evolved faster, while 48 evolved slower than the background. We scrutinised outlier genes with ω values that deviated from the rest (i.e., ω>Q3+1.5×IQR): 11 on the *Co. hominivorax* branch and 5 on *Ch. bezziana* (Table 4; Figure 3). The highest *ω* value for *Co. hominivorax* was in the gene *thin* (*ω* = 0.687, mean *ω* = 0.144) and, for *Ch. bezziana*, in the gene *lightoid* (*ω* = 0.62, mean *ω* = 0.174).

**TABLE 4:** Genes with outlying *ω* values relative to all tested genes.

Foreground species	Ortholog in *Co. hominivorax*	Annotation	*ω*
*Co. hominivorax*	g17636.t1	Thin	0.687
	g2810.t2	CG30440	0.63
	g7009.t1	GUK‐holder	0.543
	g1996.t1	Pastrel	0.5
	g20776.t1	Cytoplasmic linker protein 190	0.481
	g12713.t1	Microsomal triacylglycerol transfer protein	0.456
	g15356.t1	Rab11	0.45
	g6514.t1	Par‐1	0.448
	g18529.t1	Spinophilin	0.445
	g10778.t3	CG1399	0.436
	g1667.t1	PH4alphaSG2	0.435
*Ch. bezziana*	g1961.t1	Lightoid	0.62
	g8056.t1	CG31064	0.548
	g9623.t1	Rudhira	0.514
	g4315.t1	windei	0.503
	g3337.t1	Partner of paired	0.491

Intersecting the results (617 genes for *Co. hominivorax* and 266 for *Ch. bezziana*) revealed 61 genes (Table S15) though insufficient for GO enrichment analysis. However, analysing enriched terms separately for each species and overlapping them yielded 468 common enriched terms (Table S16), which mainly referred to broad macromolecule metabolic pathways and reproductive and developmental processes as well as stress and behaviour. Out of these 61 genes, seven were differentially expressed in the comparisons between obligate parasites and their saprophagous congeners: four in larvae and three in adult females. Notably, two of these genes (CG11190 in larvae and CG31064 in females) exhibited concordant higher expression in parasitic species.

In the branch‐site tests, we identified 548 genes for which the *bsA* model was the best fit with *Co. hominivorax* as the foreground and 245 genes with *Ch. bezziana* (Tables S17 and S18). The overlapping set included 35 genes (Figure 3; Table S19) with different codons under positive selection in each lineage. Among these, three genes in females, but none in larvae, were differentially expressed in both parasite vs. saprophagous comparisons, with two (CG16896 and CG9733) showing concordant expression. Four genes were common to branch analysis and branch‐site analyses: *Atet*, *stardust*, CG2218, and CG8740.

GO enrichment analysis of independent sets of branch‐site tests followed by overlap yielded 438 common terms (Table S20). This term set showed a similar pattern to the previous: metabolism, development, and reproduction‐related terms. However, we also identified terms related to the endocytic pathway.

## 4 Discussion

The parasitic lifestyle displayed by some Calliphoridae species is of major concern, whether for medical, veterinary, or agricultural reasons. The obligate parasites *Co. hominivorax* and *Ch. bezziana* are particularly harmful as livestock pests, causing significant economic losses in the livestock industry (Hall and Wall 1995). Thus, understanding the genetic basis of their host dependency opens up possibilities for targeted genetic interventions, such as RNA interference or gene drives, aimed at reducing their populations or preventing larval development on living hosts. Moreover, our research contributes to the broader understanding of how convergent evolution shapes complex behaviours, shedding light on similar phenomena in other parasitic organisms, including those affecting human health. To explore this, we gathered behavioural and genetic data to better understand the evolution of parasitism.

Our first question was whether the parasitic lifestyle resulted from the larvae's ability to exploit the chosen food source or the female's choice of the oviposition site. By conducting experiments measuring the larvae's survival on different substrates and the females' oviposition preferences, we confirmed previously documented species habits (dos Santos et al. 2024; Bambaradeniya et al. 2023; Muñoz‐García et al. 2016; Hall et al. 2009; Hall and Wall 1995).

Our larval survival data further elucidate the adaptive differences between obligate and facultative parasites. Both saprophagous and facultative species exhibited developmental flexibility, surviving on either fresh and warm or rotten and room‐temperature meat. *Lucilia eximia*'s high mortality rates on a fresh diet at 37°C indicate that this diet, along with elevated rearing temperatures, may not support optimal development for this species. *Lucilia eximia* larvae commonly inhabit decaying substrates associated with decomposition, aligning with their ecological role. However, infestations of living tissues do occur, though they are rare (Muñoz‐García et al. 2016). Furthermore, pupae in this experiment were kept at relatively high temperatures (37°C), which likely exceeded the cooler conditions they would experience when developing outside a host. This may explain the observed mortality, as even under laboratory conditions, *L. eximia* can successfully complete development at 26°C (Spindola et al. 2016), indicating that the 37°C environment may have exceeded their thermal tolerance for pupation.

Conversely, *Co. hominivorax* larvae failed to develop on decaying diets, even when switched from a fresh diet at 37°C, indicating stringent ecological requirements. Larval migration behaviour observed in the switch assays showed *Co. hominivorax*'s sensitivity to suboptimal conditions, possibly as a mechanism to avoid unsuitable environments. Larvae refused to eat, lost weight, and eventually died, resembling the behaviour of *Drosophila melanogaster* larvae exposed to high doses of quinine, a toxic compound (Wu et al. 2005). Our findings suggest that *Co. hominivorax*, an obligate parasite, exhibits strict dietary requirements for fresh tissue supplemented with blood at high temperature, while facultative and saprophagous species are more versatile, thriving on both fresh and rotten substrates.

The inability of *Co. hominivorax* larvae to develop on decaying meat, even when incubated at a higher temperature, contrasts starkly with the high survival rates of other species on rotten diets, reinforcing the idea of highly specialised adaptations in obligate parasitic species. This strict requirement for fresh tissue and blood at high temperatures reflects both ecological pressures and evolutionary constraints on larval development, as parasitism demands a dependable, living host. The strict host dependency of *Co. hominivorax* mirrors findings in other obligate parasites, where specialised adaptations are often required for host utilisation (Stevens and Wallman 2006).

Oviposition rates in blowflies can be highly variable across different experimental conditions. In our study, oviposition success was recorded in 40% of *Ch. megacephala*, 45% of *L. eximia* , and 50% of *Co. hominivorax* trials. Although these percentages may seem low, previous studies have reported similarly variable rates, depending on factors such as strain differences, substrate type, and environmental conditions. For *L. eximia* , Spindola et al. (2016) observed an oviposition rate of 71.6% when using non‐decomposed minced bovine meat without blood supplementation, a substrate that differs considerably from ours. For *Co. hominivorax*, Hammack (1991) reported oviposition rates between 10% and 80%, influenced by strain variation, blood concentration, and mating status. Environmental factors also play a critical role in oviposition behaviour. Temperature, in particular, imposes physiological constraints on blowfly activity and egg‐laying behaviour. Species‐specific thermal thresholds are not well characterised, but variations among species have been documented. In our experiment, all species were maintained under the same temperature conditions, which may have been suboptimal for some of them, potentially affecting oviposition rates. Moreover, in a similar experimental setup for *L. cuprina*, Cunha et al. (2023) observed no oviposition in 78% of trials, emphasising the role of individual variability, female maturation, and egg reserves in oviposition success. Given these factors, our oviposition rates are within the expected range, although individual variability, environmental conditions, and substrate differences may have contributed to the observed results.

Even with a low rate of oviposition for some species, our oviposition experiments further highlight the behavioural specialisation observed in larvae. While the saprophagous species *Co. macellaria* and *Ch. megacephala* consistently chose rotten meat at 25°C for oviposition, females of *Co. hominivorax* exhibited a strong preference for fresh meat, consistent with its status as an obligate parasite of live tissues. This behaviour aligns with the species' reliance on a specific host environment for larval development. In contrast, facultative parasites including *Ch. albiceps*, *L. cuprina*, and *L. eximia* demonstrated a broader oviposition strategy, laying eggs on both fresh and rotten substrates at different temperatures. This flexibility likely provides an advantage in variable environmental conditions, enhancing their survival and reproductive success.

The choice of oviposition site by females appears to be the primary driver of diet selection, setting the stage for larval development. Overall, our results highlight the important roles of both larvae and females in determining parasitic habits. In saprophagous species, although larvae can survive on different diets and temperatures, the strong female preference for rotten meat at 25°C ensures that larvae predominantly feed on decaying substrates. In facultatively parasitic species, the larvae's ability to use available resources at the oviposition site drives survival, given the females' lack of biased preference. For the obligate parasite *Co. hominivorax*, both the female preference for fresh substrate at 37°C and the larvae's exclusive survival on this diet align with the species' reliance on a specific host environment for larval development.

Expanding these experiments to other obligate parasites, such as *Ch. bezziana* or Australian strains of *Lucilia cuprina dorsalis*, would determine if similar patterns emerge. Since these species represent distinct transitions to obligate parasitism (Stevens and Wall 1997; Stevens 2003; Wallman et al. 2005), it remains unclear whether the same evolutionary paths led to this convergent phenotype.

With behavioural tests recovering the ecological roles of these species, we explored genetic determinants associated with the parasitic lifestyle. Both the expression and molecular evolution analyses revealed the complexity of the parasitic behaviour, involving several genes associated with functions reflecting different aspects of the transition between trophic habits, such as chemoreception, digestion, nutrient absorption, immune response, and detoxification. These include genes such as *Gr63a* (Kumar et al. 2020; McMeniman et al. 2014), *Obp19a*, *Obp56a* (Hickner et al. 2020), and *SKIP* (Tunstall et al. 2012) linked to chemosensory functions as well as genes related to feeding, digestion, and nutrient absorption like *Rab11* (Ma and Brill 2021), *PH4alphaSG2* (Abrams et al. 2006), *lightoid*, *CG31064* (Char and Pierre 2020; Gillingham et al. 2014; Kitagishi and Matsuda 2013), and *pgant5* (Tran et al. 2012). We also identified genes associated with immune response including *CG1399* (Di Prisco et al. 2013), *Tollo* (Nie et al. 2018), *Zizimin‐related* (Sampson et al. 2012), *LysE* (Tomberlin et al. 2017), *Npc2a* (Shi et al. 2012), *AttA* (Wang et al. 2010), and PGRP‐LA (Gendrin et al. 2013). Genes involved in detoxification such as *FTH1* (Tang and Zhou 2013; Cardoso et al. 2021) and response to thermal stress and preference, including *dystroglycan* (Takeuchi et al. 2009), *Hsp23*, *Hsp27* (Davis et al. 2021), thoc6, and Hpr1 (Jimeno and Aguilera 2010; Rehwinkel et al. 2004), were also detected.

Chemosensory genes play a critical role in helping females identify environmental cues when selecting an oviposition site, such as volatiles emitted by bacteria in wounds (Chaudhury et al. 2010) or CO_2_ released by a host (Kumar et al. 2020; McMeniman et al. 2014). The absence of olfactory reception has been shown to prevent females from identifying suitable substrates for egg‐laying (Paulo et al. 2021). Temperature cues might also influence this preference. One of the genes whose expression correlates positively with the female preference (i.e., it is more expressed in parasites) was *Dystroglycan*, which is involved in thermal preference in *Drosophila* (Takeuchi et al. 2009). Therefore, it would be expected that shifts in trophic habits are likely accompanied by different responses to these environmental cues.

Larvae face different challenges depending on the food source they explore. Accordingly, obligate parasites are expected to have distinct nutrition requirements, leading to differences in digestion and absorption processes compared to saprophagous species. Furthermore, carcasses and living wounds harbour distinct microbiomes that interact directly with the larvae (Tomberlin et al. 2017), thus requiring specific immune responses. These different microorganisms may also influence females, as they feed from the oviposition substrate, which provides the protein required for egg maturation (Yin et al. 1994). As a result, it is not surprising that we identified genes involved in immune response in the differential expression and correlation analyses.

Additionally, *Co. hominivorax* and *Ch. bezziana* larvae, which infest mammals, encounter unique challenges due to their host's biology. Like all vertebrates, the immune system of mammals is complex and will respond to the infestation by the larvae (Baron and Colwell 1991; Otranto 2001). Also, the constantly high body temperatures of mammals (endothermic animals) may be considered a thermal stressor for the larvae (Davis et al. 2021). Another challenge arises from the large quantities of iron present in vertebrate blood, due to haemoglobin, which can be toxic (Tang and Zhou 2013). This requires the activation of detoxification pathways, some of which have already been identified in blowflies (Cardoso et al. 2021).

Ultimately, all those functions trace back to female choice and larvae survival, which directly influence the measured behavioural phenotypes. The majority of the genes with such functions were not recovered when the results from each congeneric comparison (obligate parasite vs. non‐parasite) were overlapped. Most overlapping genes were not directly linked to these phenotypes but rather were involved in reproduction and developmental pathways, as reflected in the GO enrichment analysis. This indicates that, while some specific functions are indeed important and offer potential candidate genes for further screening, the main factors differentiating the contrasting habits within Calliphoridae appear to be more fundamental, involving broader, basal processes.

This suggests that the convergent evolution of the parasitic behaviour does not stem solely from a parallel genetic architecture. This is not entirely unexpected, particularly considering the evolutionary distance of the taxa being compared (Arendt and Reznick 2008). As with most complex traits, a more heterogeneous response is expected at lower levels of the phenotype determination, such as genes or transcripts, because they are often redundant. However, as we move up the hierarchy to pathways, functions, and the phenotype itself, the similarities tend to emerge (Elmer and Meyer 2011; Barghi et al. 2020; Lai et al. 2023). The shared enriched terms found in our comparisons—related to immune response, response to stress, metabolism, development, and reproductive processes—indicate that these pathways are essential for maintaining the fitness of obligate parasites, corroborating findings from studies on parasitism in other organisms (Poulin 2011; Techer et al. 2019). Similar to previous studies (Poulin and Randhawa 2015; Feldmeyer et al. 2017; Techer et al. 2019), we found that convergent behaviours, such as parasitism, can evolve through both parallel and non‐parallel genetic mechanisms. The identification of both concordant and antagonistic genes underscores the genetic complexity of convergence, an idea supported by Arendt and Reznick (2008) and Stern (2013).

By integrating behavioural assays with genetic analyses, we have demonstrated the pivotal roles of female oviposition preferences and larval dietary flexibility in shaping parasitic lifestyles in Calliphoridae species. Our findings also reveal the complexity of parasitism, highlighting both common and species‐specific genetic determinants. The obligate parasites *Co. hominivorax* and *Ch. bezziana* exhibit strict dietary preferences for fresh tissues at high temperatures, a trait that is reflected in both their behaviour and gene expression profiles. While some genes involved in parasitism appear to be conserved across species, others show lineage‐specific adaptations, highlighting the complexity of molecular convergence. Our findings have important implications for pest control and deepen our understanding of how convergent evolution operates at both behavioural and molecular levels. Future research should focus on expanding these experiments to other obligate parasites. Also, as new high‐quality genomes are sequenced, it will be possible to identify other candidates through analyses not yet possible, such as changes in gene family contents, which will allow exploring the genetic mechanisms in greater detail. Such efforts will enhance our understanding of the evolution of parasitism and could inform strategies to mitigate the impacts of these species on medical, veterinary, and agricultural fronts.

## Author Contributions

G.A.C. and P.M.‐M. carried out all expression and CDS analysis; G.A.C. and G.A.F. performed the behaviour assays; I.K. collected and reared *Ch. bezziana* and *Ch. megacephala* in the lab; P.J.T. maintained the fly colonies and supervised the feeding assays; T.T.T. conceived the study, acquired the financial support for the project, and coordinated the research activity planning and execution; G.A.C., P.M.‐M., and T.T.T. wrote the manuscript.

## Conflicts of Interest

The authors declare no conflicts of interest.

## Supporting information


**Figure S1.** Female oviposition site preference assay. (A) Picture of the Y‐shaped tube used in the assays. (B) Schematics of the assay. Five gravid female flies (in the bottom) were positioned in the tube and allowed to choose between two oviposition media: fresh meat at a 37°C (red circle on the left with a high‐temperature thermometer) or rotten meat at room temperature of 25°C (green circle on the right with a low‐temperature thermometer).

**Figure S2.** *Cochliomyia hominivorax* behaviour and development on rotten meat. (A) Eggs. Yellow arrow: a hatched egg, blue arrow: a dead embryo inside an egg. (B) Second instar larvae. Yellow arrows point to larvae on top of the substrate and on the container walls, suggesting an aversive behaviour towards the substrate. (C) Second instar larvae with no food in their guts. (D) For comparison with (C), third instar larvae with food in their gut, shown by the blue arrows. (E) *Cochliomyia hominivorax* developmental curve in the switch assay from the fresh diet at 37°C to rotten meat at 25°C. The average weight of 10 larvae randomly sampled every 12 h in the survival assay is shown. Error bars represent standard deviations. 36 h after hatching, the larvae were switched from the fresh diet at 37°C to rotten meat 25°C. After this point, the larvae lost weight. All larvae were dead 60 h after the eggs had hatched.

**Figure S3.** Phenotype and expression correlation. (A–L) Plots show the TPM10K‐normalised expression as a function of the logit‐transformed preference indices for each species of the 12 genes with significant correlations. In all plots, circles, triangles and squares indicate the expression value of each replicate, whilst diamonds indicate the median for the species. The vertical lines extend from the first to the third quartiles of the distribution of expression values of the replicates of each species. The black line is the regression curve whose intercept and slope were estimated by the PGLS model (see Table 4 in the main text).

Table S1.

Table S2.

Table S3.

Table S4.

Table S5.

Table S6.

Table S7.

Table S8.

Table S9.

Table S10.

Table S11.

Table S12.

Table S13.

Table S14.

Table S15.

Table S16.

Table S17.

Table S18.

Table S19.

Table S20.

## Data Availability

All raw sequencing reads are available under BioProject PRJNA1159025.
