# Supplementary figures and images for: Divergent Genetic Pathways Underlying Convergent Parasitic Behaviours in Blowflies
Source: Mol Ecol. 2025 May 5;34(11):e17785. doi: 10.1111/mec.17785 (PMC12100580; doi:10.1111/mec.17785)

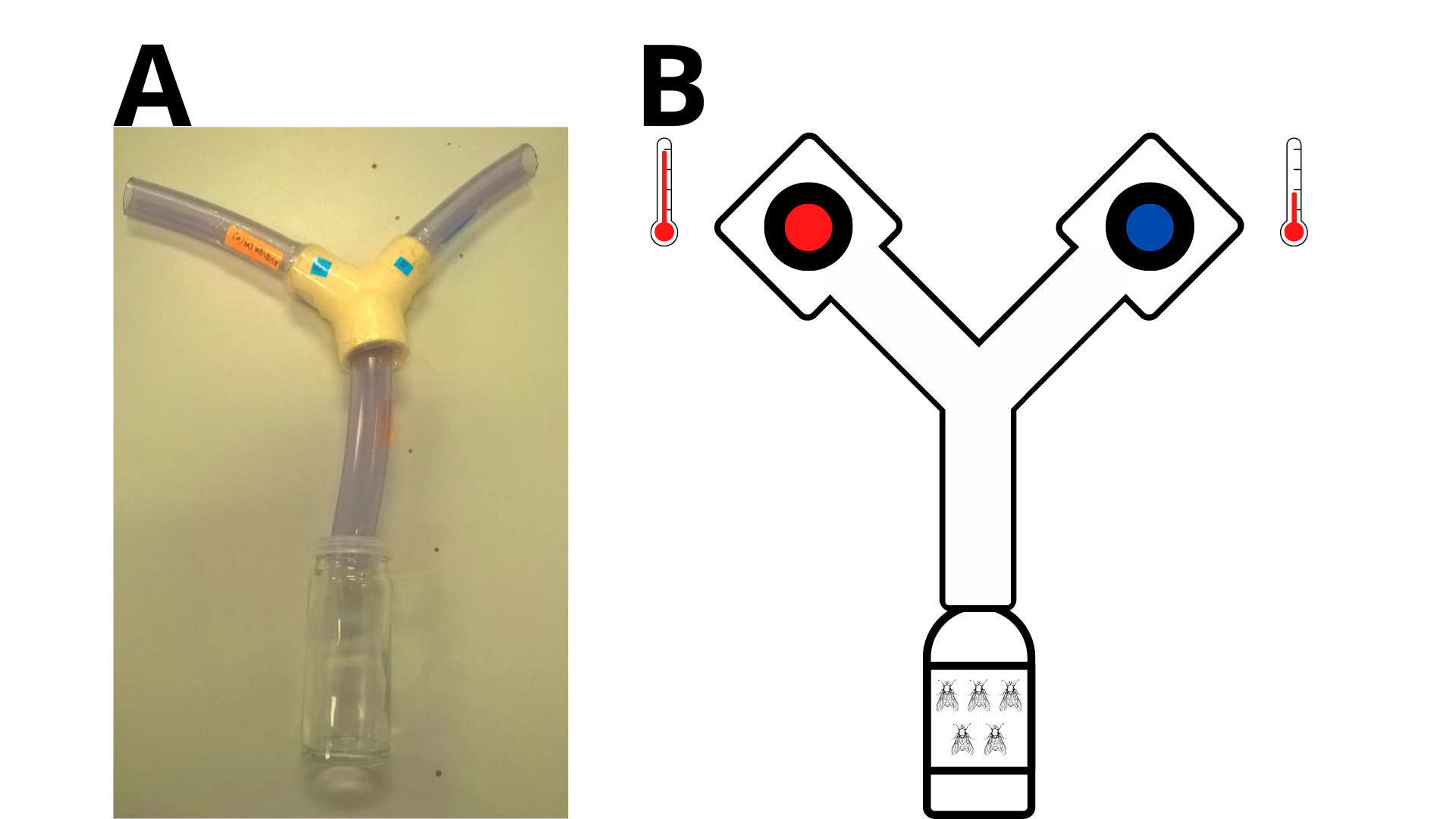

Supplement: Supplementary file 1 — Figure S1. Female oviposition site preference assay. (A) Picture of the Y‐shaped tube used in the assays. (B) Schematics of the assay. Five gravid female flies (in the bottom) were positioned in the tube and allowed to choose between two oviposition media: fresh meat at a 37°C (red circle on the left with a high‐temperature thermometer) or rotten meat at room temperature of 25°C (green circle on the right with a low‐temperature thermometer). [file MEC-34-e17785-s006.png]

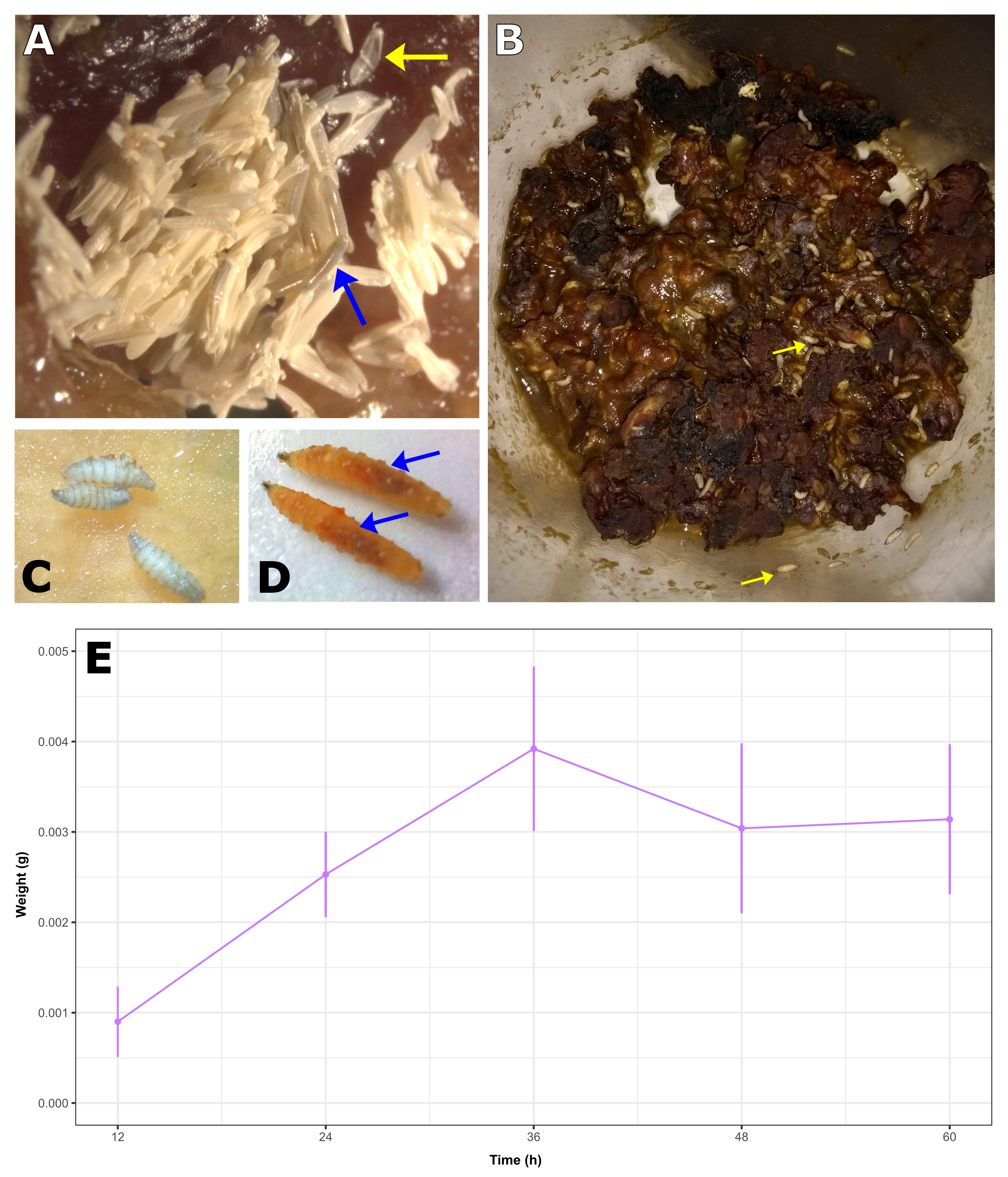

Supplement: Supplementary file 2 — Figure S2. Cochliomyia hominivorax behaviour and development on rotten meat. (A) Eggs. Yellow arrow: a hatched egg, blue arrow: a dead embryo inside an egg. (B) Second instar larvae. Yellow arrows point to larvae on top of the substrate and on the container walls, suggesting an aversive behaviour towards the substrate. (C) Second instar larvae with no food in their guts. (D) For comparison with (C), third instar larvae with food in their gut, shown by the blue arrows. (E) Cochliomyia hominivorax developmental curve in the switch assay from the fresh diet at 37°C to rotten meat at 25°C. The average weight of 10 larvae randomly sampled every 12 h in the survival assay is shown. Error bars represent standard deviations. 36 h after hatching, the larvae were switched from the fresh diet at 37°C to rotten meat 25°C. After this point, the larvae lost weight. All larvae were dead 60 h after the eggs had hatched. [file MEC-34-e17785-s009.png]

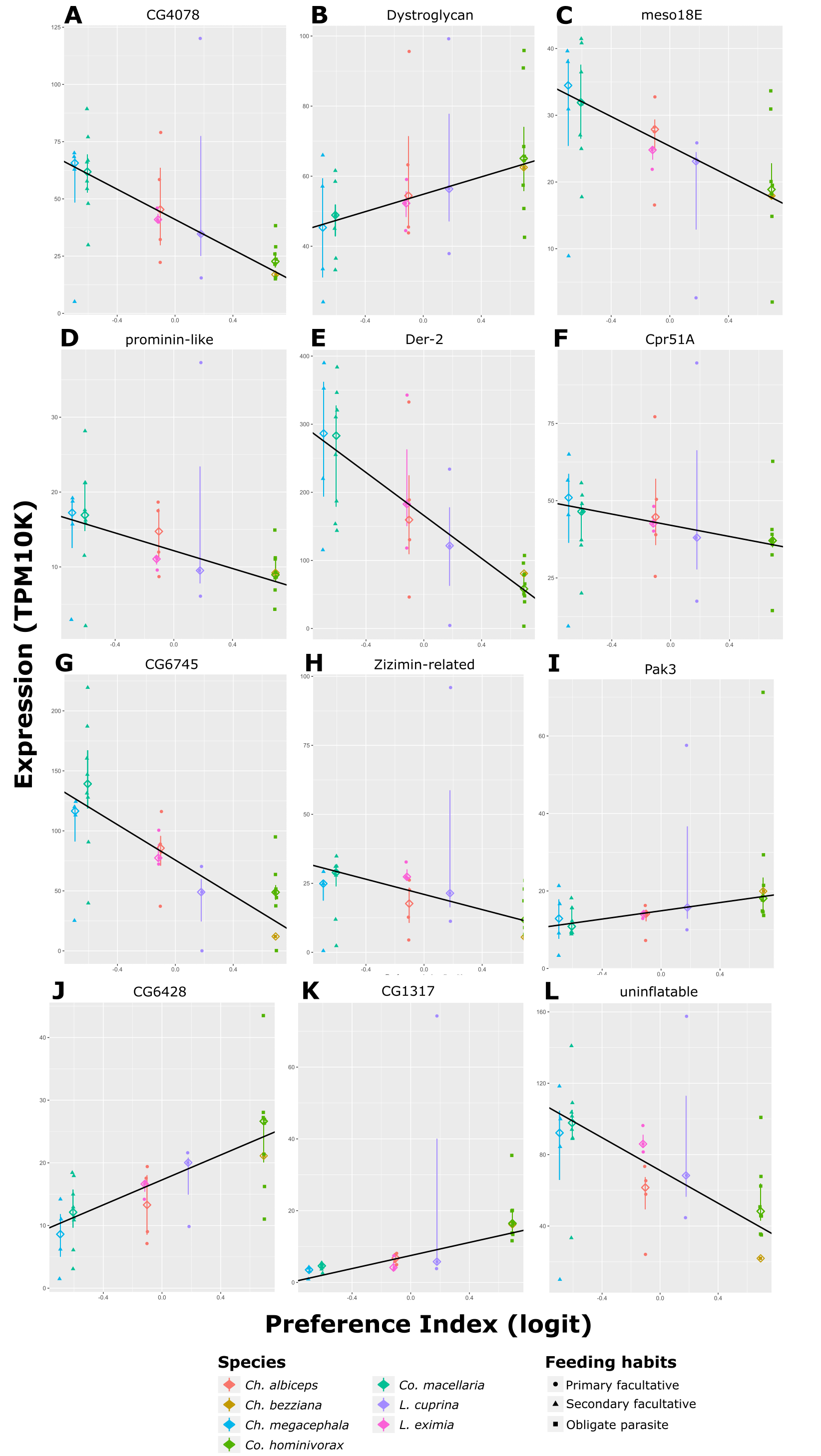

Supplement: Supplementary file 3 — Figure S3. Phenotype and expression correlation. (A–L) Plots show the TPM10K‐normalised expression as a function of the logit‐transformed preference indices for each species of the 12 genes with significant correlations. In all plots, circles, triangles and squares indicate the expression value of each replicate, whilst diamonds indicate the median for the species. The vertical lines extend from the first to the third quartiles of the distribution of expression values of the replicates of each species. The black line is the regression curve whose intercept and slope were estimated by the PGLS model (see Table 4 in the main text). [file MEC-34-e17785-s011.png]
